# The Relationship Between Retinal Nerve Fiber Layer Thickness and Clinical Symptoms of Alzheimer's Disease

**DOI:** 10.3389/fnagi.2020.584244

**Published:** 2021-01-29

**Authors:** Teng-hong Lian, Zhao Jin, Yuan-zhen Qu, Peng Guo, Hui-ying Guan, Wei-jiao Zhang, Du-yu Ding, Da-ning Li, Li-xia Li, Xiao-min Wang, Wei Zhang

**Affiliations:** ^1^Department of Neurology, Beijing Tiantan Hospital, Capital Medical University, Beijing, China; ^2^Department of Ophthalmology, Beijing Tiantan Hospital, Capital University of Medical Sciences, Beijing, China; ^3^Department of Internal Medicine, Beijing Tiantan Hospital, Capital Medical University, Beijing, China; ^4^Department of Physiology, Capital Medical University, Beijing, China; ^5^Center for Cognitive Neurology, Department of Neurology, Beijing Tiantan Hospital, Capital Medical University, Beijing, China; ^6^China National Clinical Research Center for Neurological Diseases, Beijing Tiantan Hospital, Capital Medical University, Beijing, China; ^7^Center of Parkinson's Disease, Beijing Institute for Brain Disorders, Beijing, China; ^8^Beijing Key Laboratory on Parkinson Disease, Beijing, China

**Keywords:** Alzheimer disease, retinal nerve fiber layer thickness, optical coherence tomography, clinical features, cognitive level

## Abstract

**Background/Aim:** Retinal nerve fiber layer (RNFL) thickness (RT), which can reflect the status of the retinal optic nerve cells, may be affected in patients with Alzheimer's disease (AD). There are few studies on the correlation of RT of patients with AD (AD-RT) with clinical symptoms of various cognitive domains, neuropsychiatric symptoms, and activities of daily living (ADL). This study is to investigate the relationships between RT and the abovementioned clinical symptoms of AD.

**Methods:** A total of 96 patients with AD were included in this study. RT was measured in these patients using optical coherence tomography (OCT). Demographic variables, RT, and clinical symptoms were compared between the normal and the abnormal AD-RT groups. Clinical symptoms, including cognitive symptoms, neuropsychiatric symptoms, and ADL, were evaluated using a series of rating scales.

**Results:** The relationships between RT and cognitive symptoms scores were analyzed in patients with AD. Reduced RT was found in 54.4% of patients with AD. The average RT, RT of the superior 1/2 quadrant, and RT of the inferior 1/2 quadrant of both eyes were all significantly decreased in the abnormal AD-RT group (*p* < 0.001). Overall cognitive function and performance in multiple cognitive domains, including memory, language, attention, and executive function, were also significantly impaired in the abnormal AD-RT group (*p* < 0.05). For lower RT value, the global cognitive function and the performance in multiple cognitive domains were worse. ADL was significantly compromised in patients with AD having lower RT values (*p* < 0.05).

**Conclusions:** Lower RT value appear to be correlated with cognitive impairment, and RT may be an indicator of cognitive decline in patients with AD. Further studies are required to confirm our findings.

## Introduction

The retina is a peripheral extension of the central nervous system. It has a similar tissue source and an anatomical structure to the central nervous system, including neurons, ganglion cells, and a blood barrier (Maccormick et al., 2015; Trost et al., 2016; Diaz-Coranguez et al., 2017). The typical pathological hallmarks of Alzheimer's disease (AD), including the accumulation of amyloid plaques and neurofibrillary tangles, can affect the relevant regions of the visual cortex in the early stage of the disease (Sperling et al., 2011). Retinal nerve fiber layer (RNFL) thickness (RT), which can reflect the status of the retinal optic nerve cells, may also be affected in patients with AD. Moreover, degeneration in the RNFL has been shown to parallel disease severity in patients with AD (Liu et al., 2015; Garcia-Martin et al., 2016). Research has shown that RT is lower in patients with AD than in normal elderly individuals (Gao et al., 2015). Interestingly, RNFL thinning has been observed in the early stage of dementia and the mild cognitive impairment (MCI) stage of patients with AD, suggesting the potential value of RNFL thinning in the early identification of AD (Holroyd and Shepherd, 2001). Some studies have not found a significant association of retinal thinning with lower Mini–Mental State Examination (MMSE) score (Cipollini et al., 2020), while other studies showed a significant correlation between the MMSE score and the RNFL value (Oktem et al., 2015). Studies reported the correlation between RT and the decline of the overall cognitive function, indicating that the thinner the RT, the worse the overall cognitive function (Tzekov and Mullan, 2014; Cheung et al., 2015). Research found that the reduction in the magnitude of macular RNFL volume significantly correlated with the performance of participants' abilities on a task to efficiently integrate visual and auditory speech information (Santos et al., 2018). A study on a few samples showed that RT correlated with the neuropsychological performance in multiple cognitive domains (e.g., working memory, psychomotor speed, and executive function; Mammadova et al., 2020). However, there are a few studies that have examined the correlation between RT in patients with AD (AD-RT) and various cognitive domains, including memory, attention, language, visuospatial ability, and executive function, as well as neuropsychiatric symptoms, such as anxiety, depression, and agitation.

In this study, RT was measured using optical coherence tomography (OCT), and the clinical characteristics of AD-RT were analyzed. Demographic variables were collected and clinical symptoms, including cognitive function, neuropsychiatric symptoms, and activities of daily living (ADL), were evaluated using a series of rating scales. The relationships between RT and demographic variables and clinical symptoms of patients with AD were analyzed. This investigation aimed to provide a clinical basis for understanding the changes of RT in patients with AD and its correlation with the clinical symptoms of AD.

## Materials and Methods

### Ethics Statement

This study met the guidelines on ethical principles for medical research involving human subjects of the Declaration of Helsinki, and the study protocol was approved by the Ethical Review Board of Beijing Tiantan Hospital. Written informed consent was obtained from patients and their family members. All methods were performed following relevant guidelines and regulations.

### Subjects

#### Inclusion Criteria

This study included patients with mild cognitive impairment (MCI) due to AD (Albert et al., 2011) and patients with AD dementia (Mckhann et al., 2011) according to the National Institute of Aging and Alzheimer's Association (NIA-AA) criteria.

#### Exclusion Criteria

The exclusion criteria of this study were as followed: (1) the presence of one or more of the following ophthalmic diseases: glaucoma, cataract, optic neuropathy, retinal vascular disease, retinal detachment, and macular degeneration; (2) the presence of high myopia >600°, pupil <2 mm; (3) a history of ocular trauma; (4) previous ophthalmological surgeries performed within the previous 6 months; (5) the presence of systemic diseases, including hypothyroidism, severe chronic diseases, and other medical diseases that might affect vision; and (6) a history of alcoholism or carbon monoxide poisoning.

#### Collection of Demographic Information

Demographic variables, including gender, age, age of onset of AD, disease duration, and education level, were recorded for all participants with AD.

### Evaluation of RT by OCT

The patient was seated in a quiet state and scanned using an RTVue 100 OCT machine (Optovue, Inc., Fremont, CA, USA), with the optic nerve head (ONH) scanning mode. Optic nerve head-centered circular scanning was performed at a depth of 5 μm and a diameter of 3.45 mm. The scanning proceeded through each quadrant in turn. The right eye was scanned clockwise, while the left eye was scanned counter-clockwise. RT value was generated after automatic mapping and evaluation using a computer program. Ganglion cell complex thickness (GCCT) of each eye was also evaluated. Repeated scans were performed three times, and the average value was taken as AD-RT. Both eyes were scanned by the same ophthalmologist who did not know the patient's diagnosis, and the images and results were saved.

The measured quadrants included the superior nasal (SN), nasal upper (NU), nasal lower (NL), inferior nasal (IN), inferior temporal (IT), temporal lower (TL), temporal upper (TU), and superior temporal (ST) quadrants, in both oculus dexter (OD), and oculus sinister (OS). The superior 1/2 quadrants included the SN, NU, TU, and ST quadrants, and the inferior 1/2 quadrants included the NL, IN, IT, and TL quadrants. After scanning the eyes of the patients with AD using OCT, the computer image analysis system compared the RT values obtained with the normal values in the database for the same age, sex, eye, and part, and color-coded it green, yellow, and red to represent the confidence intervals of 95–5%, 5–1%, and 1–0% of the normal population, respectively (Supplemantery 1).

### Assessment of Cognitive Function

#### Global Cognitive Function

Mini-mental State Examination (MMSE) scale was used to evaluate the global cognitive function of patients with AD. The lower the score of the MMSE scale, the more severe the cognitive impairment.

Cognitive impairment was established in patients with illiteracy, primary education, and junior and higher education when the MMSE score was below 17, 20, and 24 points, respectively. It is one of the standards for the Chinese version of MMSE, which cutoff value was formulated by Zhang (1995).

Individual cognitive domains were assessed using the following rating scales:

##### Memory

Visual delayed memory was evaluated by the Rey-Osterreith Complex Figure Test (CFT)-delayed memory (Guo et al., 2009). Low score of this scale indicated poor verbal and visual memories.

##### Visuospatial Ability

Visuospatial ability was evaluated using the CFT-imitation (Guo et al., 2009). A low score in this test suggested worse visuospatial ability.

##### Language

Language function was evaluated using the Animal Fluency Test (AFT; Lin et al., 2014). A low score implied compromised language function.

##### Attention

Attention was evaluated using the Trail Making Test A (TMT-A;Wei et al., 2018). The longer it took to complete the test, the worse an individual's attention.

##### Executive Function

Executive function was rated using the Stroop Color-Word Test (SCWT;Bondi et al., 2002). A low score of this test indicated an impaired executive function.

#### Assessment of Neuropsychiatric Symptoms

Overall neuropsychiatric symptoms were assessed using the Neuropsychiatric Inventory (NPI). A high score implied severe overall neuropsychiatric symptoms.

Individual neuropsychiatric symptoms were then assessed using the following rating scales:

##### Depression

Depression was evaluated using the Hamilton Depression Scale (HAMD)-24 items. Higher score indicated more severe depression, and a score of ≥8 confirms the presence of depression.

##### Anxiety

Anxiety was evaluated using the Hamilton Anxiety Scale (HAMA)-14 items. An elevated score of this test suggested more severe anxiety, and a score of ≥8 confirmed the presence of anxiety.

##### Agitation

Agitation was rated using the Cohen-Mansfield Agitation Inventory (CMAI). The higher the CMAI score, the more severe the agitation.

##### Apathy

Apathy was rated using the Modified Apathy Estimate Scale (MAES). The higher the score, the more severe the apathy. A score of >14 indicated clinically meaningful apathy.

#### Assessment of the Activities of Daily Living

Activities of daily living were assessed using the ADL scale, which includes the basic (ADL) (BADL) and the instrumental ADL (IADL) scales. The higher the score, the worse the ADL performance (Zhang, 1995).

### Data Analyses

Statistical analyses were performed using the SPSS Statistics 20.0 (IBM Corp., Armonk, NY, USA). A value of *p* < 0.05 was considered statistically significant.

Continuous variables, if normally distributed, are presented as means ± SDs and were compared using the two-sample *t*-test. Continuous variables, if they were not normally distributed, are presented as medians (quartiles) and were compared using a non-parametric test. The bivariate correlation method was used to analyze the correlation of the measurement data.

Demographic variables, RT, and clinical symptoms were compared between the normal and the abnormal AD-RT groups. Clinical symptoms, including cognitive symptoms, neuropsychiatric symptoms, and ADL determined by the corresponding rating scales, were compared between the normal and the abnormal AD-RT groups.

Spearman correlation analyses were performed between the RT and the scores of cognitive symptoms, neuropsychiatric symptoms, and ADL in patients with AD.

## Results

### The Frequency of Abnormal RT in Patients With AD

Of the 96 patients with AD included in this study, 52 (54.17%) had an abnormal RT and 44 (45.83%) had a normal RT, as revealed by OCT.

### Comparison of RT Between the Normal and the Abnormal AD-RT Groups

The RTs of the right and left eyes in the normal and the abnormal AD-RT groups were compared. Compared to the normal AD-RT group, the average RT, the RT of the superior 1/2 quadrants, and the RT of the inferior 1/2 quadrants of the right and left eyes were all significantly decreased in the abnormal AD-RT group (*p* < 0.001; Table 1). The average GCCT, the GCCT of superior 1/2 quadrant, and the GCCT of the inferior 1/2 quadrant of left eye were all significantly decreased in the abnormal AD-RT group (*p* < 0.05; Table 1).

**Table 1:** Comparison of retinal nerve fiber layer (RNFL) thickness (RT) and ganglion cell complex thickness (GCCT) between the normal and the abnormal RT of patients with AD (AD-RT) groups.

	**Normal AD-RT group** **(44 cases)**	**Abnormal AD-RT group** **(52 cases)**	***p***
**Right eye**			
Average RT [μm,	109.82 (101.50, 115.53)	95.25 (91.06, 103.94)	<0.001^**^
median (quartile)]			
RT of the superior 1/2 quadrant	109.86 ± 10.76	97.40 ± 12.97	<0.001^**^
(μm, mean ± SD)			
RT of the inferior 1/2 quadrant	107.85 ± 11.27	95.76 ± 11.73	<0.001^**^
(μm, mean ± SD)			
**Left eye**			
Average RT (μm, mean ± SD)	109.26 ± 9.37	96.70 ± 9.39	<0.001^**^
RT of the superior 1/2 quadrant	112.09 ± 10.16	99.09 ± 12.06	<0.001^**^
(μm, mean ± SD)			
RT of the inferior 1/2 quadrant	107.49 (99.29, 112.47)	94.05 (87.79, 99.11)	<0.001^**^
[μm, median (quartile)]			
**Right eye**			
Average GCCT (μm, mean ± SD)	91.07 ± 15.95	87.3 ± 8.13	0.144
GCCT of the superior 1/2	91.05 ± 16.06	87.82 ± 9.29	0.228
quadrant (μm, mean ± SD)			
GCCT of the inferior 1/2	90.86 ± 16.02	85.22 ± 13.66	0.069
quadrant (μm, mean ± SD)			
**Left eye**			
Average GCCT (μm, mean ± SD)	92.77 ± 8.56	84.92 ± 20.56	0.022^*^
GCCT of the superior 1/2	92.42 ± 8.15	84.92 ± 20.31	0.026^*^
quadrant (μm, mean ± SD)			
GCCT of the inferior 1/2	93.07 ± 9.45	83.74 ± 21.38	0.01^*^
quadrant (μm, mean ± SD)			

** *p < 0.01*,

* *p < 0.05*.

### Relationship Between RT and Demographic Variables

Demographic variables, including sex, age, age of onset, disease duration, and education level were compared between the normal and the abnormal AD-RT groups. No significant differences in the demographic variables were observed between the two groups (Table 2).

**Table 2:** Comparisons of demographic variables between the normal and the abnormal AD-RT groups.

	**Normal AD-RT group** **(44 cases)**	**Abnormal AD-RT group** **(52 cases)**	***p***
**Sex**			
Male [cases (%)]	16 (36.36%)	23 (44.23%)	0.434
Female [cases (%)]	28 (63.64%)	29 (55.77%)	
Age (years, mean ± SD)	65.98 ± 9.307	69.6 ± 10.01	0.078
Age of onset (years, mean ± SD)	63.43 ± 9.195	65.07 ± 12.005	0.479
Disease duration [years, median (quartile)]	3.00 (2.00, 4.00)	3.00 (2.00, 5.00)	0.144
**Education level [cases (%)]**			
Primary school and below	11 (25.00%)	13 (25.00%)	0.213
Middle and high school	18 (40.91%)	29 (55.77%)	
Bachelor's degree and above	15(34.09%)	10 (19.23%)	

### Relationship Between RT and Cognitive Function

First, the relationship between RT and overall cognitive function was analyzed. The MMSE score in the abnormal AD-RT group was significantly lower than that in the normal AD-RT group (*p* < 0.01; Table 3).

**Table 3:** Comparison of cognitive function between the normal and the abnormal AD-RT groups.

	**Normal AD-RT group** **(44 cases)**	**Abnormal AD-RT group** **(52 cases)**	***p***
**Global cognitive function**			
MMSE [scores, median (quartile)]	25.00 (18.00, 29.00)	21 (14.00, 26.00)	0.036^*^
**Cognitive domain**			
**Memory**			
CFT-delayed memory [scores, median (quartile)]	3.00 (0.00, 10.00)	0.00 (0.00, 4.50)	0.032^*^
**Language**			
AFT [scores, median (quartile)]	14.00 (9.00, 20.00)	9.5 (6.00, 14.75)	0.003^*^
**Attention**			
TMT-A-time [seconds, median (quartile)]	82.00 (60.00, 125.50)	129.00 (66.00, 220.50)	0.033^*^
**Visuospatial ability**			
CFT-imitation [scores, median (quartile)]	22.00 (6.25, 34.00)	14.00 (2.00, 28.00)	0.122
**Executive function**			
SCWT-time [seconds, median (quartile)]	77.00 (64.50, 105.00)	110.00 (74.00, 161.00)	0.006^*^

* *p < 0.05*.

*MMSE, Mini-Mental State Examination; MoCA, Montreal Cognitive Assessment; CFT, Complex Figure Test; AFT, Animal Fluency Test; TMT, Trial Making Test; SCWT, Stroop Color Word Test*.

The correlation between RT and MMSE score was then analyzed. The results indicated that the average RT and the RT of the superior 1/2 quadrant of the right eye were significantly and positively correlated with the MMSE score in patients with AD (Table 4), indicating that in patients with AD, the lower the RT value, particularly the RT of the superior 1/2 quadrants of the right eye, the worse the overall cognitive function (Table 4).

**Table 4:** Correlation analysis between RT and global cognitive function of patients with AD.

	**Score of MMSE scale**	
	**R**	***p***
**Right eye**		
Average RT	0.232	0.023^*^
RT of superior 1/2 quadrant	0.261	0.001^*^
RT of inferior 1/2 quadrant	0.144	0.163
**Left eye**		
Average RT	0.147	0.153
RT of superior 1/2 quadrant	0.185	0.070
RT of inferior 1/2 quadrant	0.045	0.660

* *p < 0.05*.

Secondly, the relationship between the RT and individual cognitive domains was analyzed.

#### Memory

The CFT-delayed memory score in the abnormal AD-RT group was significantly lower than that in the normal AD-RT group (Table 3), indicating that the visual delay recall function of the abnormal AD-RT group was significantly compromised when compared with the normal AD-RT group (*p* < 0.01; Table 3). The average RT and the RT of the superior 1/2 quadrant of the right eye were significantly and positively correlated with the CFT-delayed memory score in patients with AD (Table 5), illustrating that the lower the RT value, particularly the RT of the superior 1/2 quadrant of the right eye, the more severe the visual delayed recall impairment in patients with AD.

**Table 5:** Correlation analysis between RT and individual cognitive domain in patients with AD.

	**Score of** **CFT-delayed memory**		**Score of AFT scale**		**Time used for TMT-A**		**Time used for SCWT**	
	**r**	***p***	**r**	***p***	**R**	***p***	**r**	***p***
**Right eye**								
Average RT	0.385	0.001^**^	0.294	0.004^**^	−0.053	0.634	−0.152	0.200
RT of superior 1/2 quadrant	0.368	0.002^**^	0.298	0.003^**^	−0.132	0.236	−0.203	0.085
RT of inferior 1/2 quadrant	0.310	0.009^**^	0.230	0.024^*^	0.045	0.691	−0.07	0.555
**Left eye**								
Average RT	0.308	0.010^*^	0.271	0.008^**^	−0.083	0.457	−0.222	0.059
RT of superior 1/2 quadrant	0.337	0.005^**^	0.237	0.020^*^	−0.158	0.156	−0.182	0.123
RT of inferior 1/2 quadrant	0.224	0.065	0.209	0.041^*^	−0.001	0.991	−0.238	0.042^*^

* *p < 0.05*,

** *p < 0.01*.

#### Language

The AFT score in the abnormal AD-RT group was significantly lower than that in the normal AD-RT group (Table 3), demonstrating that the language function of the abnormal AD-RT group was significantly damaged. The AFT scale score was significantly and positively correlated with the average RT, the RT of the superior 1/2 quadrants, and the RT of the inferior 1/2 quadrants of the right eye in patients with AD (Table 5), demonstrating that in patients with AD, the lower the RT value of the right eye, the worse the language function.

#### Attention

The time taken to complete the TMT-A in the abnormal AD-RT group was significantly longer than that in the normal AD-RT group (*p* < 0.01; Table 3), illustrating that attention in the abnormal AD-RT group was markedly impaired relative to that in the normal AD-RT group.

#### Executive Function

The time taken to complete the SCW scale in the abnormal AD-RT group was significantly longer than that in the normal AD-RT group (*p* < 0.01; Table 3), illustrating that the executive function in the abnormal AD-RT group was significantly damaged relative to that in the normal AD-RT group. The time taken to complete the SCW scale was significantly and negatively correlated with the average RT, the RT of the superior 1/2 quadrant, and the RT of the inferior 1/2 quadrant of the left eye (Table 5), implying that, in patients with AD, the lower the RT of the left eye, the worse the executive function.

#### Visuospatial Ability

There were no significant differences in the Rey Complex Figure Test (RCFT) and the Clock Drawing Test (CDT) scores between the normal and the abnormal AD-RT groups (Table 3), demonstrating that RT was unrelated to the visuospatial ability in patients with AD in our study.

### Relationship Between RT and Neuropsychiatric AD Symptoms

First, the relationship between the RT and the total neuropsychiatric symptoms was analyzed. There was no significant difference in the NPI score between the normal and the abnormal AD-RT groups (Table 6), demonstrating that the RT was not related to the total neuropsychiatric of AD symptoms.

**Table 6:** Comparisons of neuropsychiatric symptoms between the normal and the abnormal AD-RT groups.

	**Normal AD-RT group** **(44 cases)**	**Abnormal AD-RT group** **(52 cases)**	***p***
NPI [scores, median (quartile)]	1.50 (0.00, 8.25)	1.00 (0.00, 4.00)	0.996
HAMD [scores, median (quartile)]	4.50 (2.00, 9.00)	7.77 ± 7.29	0.172
HAMA [scores, median (quartile)]	4.00 (1.25, 6.00)	5.00 (2.00, 10.00)	0.110
CMAI [scores, median (quartile)]	29.00 (29.00, 29.00)	29.00(29.00, 29.00)	0.498
MAES (scores, mean ± SD)	12.66 ± 10.94	16.89 ± 12.52	0.094

*NPI, Neuropsychiatric Inventory; HAMD, Hamilton Depression Scale; HAMA, Hamilton Anxiety Scale; CMAI, Cohen-Mansfield Agitation Inventory; MAES, Modified Apathy Evaluation Scale*.

Second, the relationship between the RT and the individual neuropsychiatric symptoms were analyzed. There were no significant differences in the HAMD, the HAMA, the CMAI, and the MAES scale scores (Table 6), demonstrating that the RT was not related to depression, anxiety, agitation, or apathy in patients with AD.

### Relationship Between RT and ADL

We compared the ADL scale scores between the normal and the abnormal AD-RT groups, and the results demonstrated that the ADL score in the abnormal AD-RT group was significantly higher than that in the normal AD-RT group [26.00 (20.00, 37.00) vs. 20.00 (20.00, 25.00), *p* < 0.01], illustrating that the ADL score in the abnormal AD-RT group was significantly lower than that in the normal AD-RT group. The ADL score was significantly and negatively correlated with the average RT of the right eye in patients with AD (Table 7), demonstrating that the lower the average RT values of the right eye, the worse the ADL score of patients with AD.

**Table 7:** Correlation analysis of RT and ADL in patients with AD.

	**Score of ADL scale**	
	**r**	***p***
**Right eye**		
Average RT	−0.383	<0.001^**^
RT of superior 1/2 quadrant	−0.410	<0.001^**^
RT of inferior 1/2 quadrant	−0.275	0.011^*^
**Left eye**		
Average RT	−0.273	0.012^*^
RT of superior 1/2 quadrant	−0.328	0.002^*^
RT of inferior 1/2 quadrant	−0.128	0.246

* *p < 0.05*,

** *p < 0.01*.

## Discussion

Abnormal RT has not attracted an extensive attention from clinicians or patients with AD. Therefore, the frequency of abnormal AD-RT has rarely been reported. In this study, OCT was used to measure the RT of patients with AD. According to the diagnostic criteria adjusted for sex and age, the results demonstrated that the frequency of the abnormal AD-RT was 54.16%. The RT can be influenced by many factors, for example, age and vascular risk factors (Gattoussi et al., 2019). It appears to be correlated with cognitive impairment, which was shown in our study. In the present study, there were 44 cases (45.83%) of all AD patients with normal RT, which was speculated that normal AD-RT group had higher global cognitive function.

At present, there is no unified conclusion in the study of RT among patients with AD. The RT of the superior 1/4 quadrant (ST+SN regions) and the inferior 1/4 quadrant (IT+IN regions) have been shown to be reduced in patients with AD, while no significant changes were observed in other retinal areas (Lu et al., 2010). It has also been shown that the average, superior, and inferior RT were significantly decreased in patients with AD (Ngoo et al., 2019). The global and temporal superior quadrants' peripapillary RNFL and the superior pericentral and peripheral sectors of the overall RT in patients with AD have been shown to be significantly thinner (Cunha et al., 2017). In this study, the RT of the superior 1/2 quadrant and of the inferior 1/2 quadrant and the average RT of each eyes were all significantly decreased. Studies have found that the decrease in RT in the superior 1/2 quadrant in patients with AD might be caused by the following reasons: the axons of the RNFL in the superior 1/2 quadrant reach the cuneus gyrus of the primary optic cortex through the optic radiation, and the axons of the RNFL in the inferior 1/2 quadrant reach the lingual gyrus through the optic radiation. It has been reported that the densities of the neuroinflammatory response in amyloid plaques and neurofibrillary tangles in the cuneus gyrus are significantly higher than those in the lingual gyrus; therefore, the RT of the superior 1/2 quadrants was more prone to be thinner (Armstrong, 1996). Additionally, we observed that the RT of the inferior 1/2 quadrant was significantly decreased; thus, the average RT was decreased because both the superior 1/2 and inferior 1/2 quadrants were reduced. It has been suggested that visual abnormalities in patients with AD might be associated with neurodegeneration in the visual cortex, and the neuroinflammatory response in amyloid plaques as well as the neurofibrillary tangles might occur in the visual cortex in the early stage of AD—even earlier than in the hippocampus (Mckee et al., 2006). Amyloid precursor protein (APP) is synthesized in retinal ganglion cells and rapidly transported into the optic nerve in small transport vesicles (Morin et al., 1993). Combined with the cognitive decline in the abnormal AD-RT group, we speculated that there might be more β amyloid (Aβ) deposition and more severe neurodegeneration. Thus, the RT of the superior 1/2 quadrant and of the inferior 1/2 quadrant and the average RT of both eyes might be dramatically reduced in the abnormal AD-RT group.

The relationship between RT and demographic variables was analyzed between the normal and the abnormal AD-RT groups. Similar to other studies, we failed to find a significant gender difference between the two groups. An early study reported that RT decreased with age (Balazsi et al., 1984), while another study reported a minimal effect of age on RT (Polito et al., 2002). A recent study showed that the RT of some sectors decreased with age (Jang et al., 2018), and age was not a constant confounder when using OCT (Hsu et al., 2012). We found no differences in age or age of onset between the two groups. Over time, Aβ and tau in the retina of patients with AD might gradually accumulate, and RT might progressively reduce. However, similar to other investigations, we did not observe different disease durations between the two groups. No previous study has focused on RT and the education level; here, we did not observe a difference in the education levels between the two groups. In conclusion, there were no differences in the abovementioned demographic variables between the two groups, indicating no relationship between RT and demographic variables.

The impaired cognitive domains in patients with AD mainly included memory, attention, language, visuospatial ability, and executive function.

The MMSE scale covers multiple cognitive domains and has high sensitivity for evaluating dementia and MCI. In this study, the overall cognitive function was assessed using the MMSE scale. The results demonstrated that the score of MMSE scale in the abnormal AD-RT group were significantly lower than those of the normal AD-RT group, and the average RT and the RT of the superior 1/2 quadrant of the right eye in patients with AD were significantly and positively correlated with the score of the MMSE scale; this illustrated that the overall cognitive function of the abnormal AD-RT group was dramatically impaired, and the lower the RT value, the more severe the cognitive decline in patients with AD.

Some studies have shown a significant correlation between the RT and the MMSE score (Iseri et al., 2006; Oktem et al., 2015), indicating that RT reduced with the aggravation of AD. While some investigations failed to find a significant association between retinal thinning and decreased MMSE score (Cipollini et al., 2019), this study showed that the MMSE score in the abnormal AD-RT group was dramatically decreased compared to the normal AD-RT group, suggesting that the lower the RT value, the worse the cognitive function. A previous study showed that intravenous immunoglobulin might reduce Aβ depositions in retina and central nervous system (Kile et al., 2020). We speculated that the depositions of pathological proteins in the AD brain were increased, causing severe neuronal damage and subsequent overall cognitive impairment. Aβ and tau might accumulate in the retinas of patients with AD, progressively causing RT reduction.

Complex Figure Test-delayed memory is a commonly used rating scale for visual memory (Siri et al., 2001). Nonverbal spatial memory and imitation are often neglected in the clinical examination. Impairment of visual memory is one of the most important early manifestations of AD (Hayashi et al., 2018; Oltra-Cucarella et al., 2018). There has been no prior study evaluating the relationship between RT and CFT-delayed memory in patients with AD. In this study, the CFT-delayed memory score in the abnormal AD-RT group was significantly decreased, demonstrating that the visual delayed memory was dramatically damaged in the abnormal AD-RT group. In addition, the average RT and the RT of the superior 1/2 quadrant of the right eye were significantly and positively correlated with the CFT-delayed memory score, illustrating that the lower the RT value, the more severe the visual delayed memory impairment in patients with AD. Thus, the visual memory of patients with AD might be obviously affected by the reduction of RT, resulting in the visual delayed memory being dramatically impaired in the abnormal AD-RT group.

A verbal fluency test was used to investigate the relationship between RT and language impairment in patients with AD. Verbal fluency reflects instant verbal memory, spontaneous verbal motor ability, interference suppression ability, and thinking organization ability. In this study, the verbal fluency test required patients to list as many animal names as possible within 1 min. We found that the language function was dramatically damaged in the abnormal AD-RT group. Moreover, the average RT and the RT of the superior and the inferior 1/2 quadrants of the right eye were significantly and positively correlated with the score of verbal fluency test. AD is a neurodegenerative disease that extensively affects the cerebral cortex bilaterally; however, Aβ may form in different brain regions in the early stages, manifesting as different clinical types with different initial symptoms. Thus, language may be impaired when the lesions affect language-related regions of the brain. Patients with AD and amnestic MCI showing left-dominant hypometabolism tend to present severe impairment in verbal memory and be diagnosed with AD dementia (Murayama et al., 2016). Moreover, better cognitive performance has been shown to be significantly associated with increased RT in all tests, including a semantic fluency (animals and professions) test (Van Koolwijk et al., 2009). This study found evidence of an obvious language impairment in the abnormal AD-RT group. We speculated that the decrease in RT might compromise the transmission of retinal information to the occipital striate region, the primary visual cortex, which has extensive fibrous connections with the bilateral cerebral hemispheres that link the visual information to language processing. This might explain why the language was profoundly impaired in the abnormal AD-RT group.

The Trail Making Test A was used to explore the relationship between RT and attention in patients with AD. In this study, the time taken to complete the TMT-A in the abnormal AD-RT group was significantly prolonged, suggesting that attention was markedly impaired in this group. A previous study found that patients with AD had attention impairment in the early stage of the disease. Attention might be the second impaired cognitive domain after memory in patients with AD, manifesting earlier than the impairments in language and visuospatial ability (Perry et al., 2000). No previous study has examined the relationship between RT and attention in patients with AD. Optimal attention performance depends on the dorsal attention network (DAN) and the ventral attention network (VAN). DAN and VAN employ the dorsal frontoparietal areas (including the intraparietal sulcus, the frontal eye fields, etc.) and ventral frontoparietal areas (including the right-lateralized temporoparietal junction and ventral frontal cortex), respectively (Corbetta and Shulman, 2002; Fox et al., 2006). The brain regions associated with RT abnormalities include the temporal and occipital lobes. Accordingly, we speculated that there was an overlap between the regions related to attention impairment and abnormal RT, which might be the potential anatomical basis for attention impairment in the abnormal AD-RT group.

Complex Figure Test-imitation was used to investigate the relationship between RT and the visuospatial ability in patients with AD. Visuospatial impairment was a prominent early feature of clinically probable AD (Mandal et al., 2012). It has ventral and dorsal pathways. The ventral pathway starts from the occipital lobe and projects to the lower temporal cortex, which is mainly responsible for perceiving and identifying the objects seen by the eyes, as well as for the storage of the visuospatial memory in the medial temporal lobe and the hippocampus. The dorsal pathway also starts from the occipital lobe and projects to the parietal lobe, the prefrontal lobe, the cortical anterior motor region, and the medial temporal lobe, participating in the formation of visuospatial memory, visual navigation, and the subsequent processing of the visuospatial ability (Tales et al., 2005, 2011). Previous studies have demonstrated that patients with AD have visuospatial impairment (Yaari and Corey-Bloom, 2007; Mendez et al., 2018), but there has been no published research on the relationship between RT and the visuospatial ability. In this study, the two groups did not differ in visuospatial ability, which needs further investigation in large samples.

The Stroop Color-Word Test was used to evaluate the relationship between RT and the executive function in patients with AD. To the best of our knowledge, there has been no previous study on the relationship between AD-RT and executive impairment. In this study, the time taken to complete the SCWT in the abnormal AD-RT group was significantly prolonged, and the average RT and the RT of the superior and the inferior 1/2 quadrants of the left eye were significantly and negatively correlated with the time taken to complete the SCWT, illustrating that AD-RT was correlated with executive function. Executive function is an important cognitive domain that represents the control and processing capability of advanced behavior. Executive dysfunction in AD included poor selective and divided attention, failed inhibition of interfering stimuli, and poor manipulation skills (Kirova et al., 2015). Executive function was mainly regulated by the frontal lobe (Alvarez and Emory, 2006). When eyes receive visual stimulation and transmit visual information from the optic nerve cells in the retina to the visual center, the whole visual neural network is activated between the primary visual cortex (occipital lobe) and the secondary cortex (the prefrontal lobe, the parietal lobe, the temporal lobe, spindle gyrus, and orbitofrontal gyrus). Lesions in the frontal lobe, a brain region that is vulnerable to AD, might destroy the visual neural network, which might be one of the reasons why the abnormal AD-RT group had executive impairment.

A body of neuropsychiatric symptoms in AD compromise the quality of life of a patient. Here, we analyzed the relationship between AD-RT and neuropsychiatric symptoms. The two groups did not differ in anxiety, depression, agitation, and apathy symptoms. Apathy was one of the commonest neuropsychiatric symptoms of AD, and significantly impaired the ADL of patients and increased the burdens of caregivers. (Sultzer, 2018). The structural integrity of the left anterior cingulate gyrus, the posterior cingulate gyrus, and the splenium, trunk and genu of the corpus callosum in patients with AD and apathy have been shown to be absent and significantly correlated with the severity of apathy (Hahn et al., 2013). We did not observe a correlation between RT and apathy, which might be due to different brain areas involved in apathy and reduced RT. A previous study reported that anxiety and depression in patients with AD were associated with a decreased metabolism in the parietal lobe. The subjective symptoms of depression were associated with high metabolism in the frontal lobe and low metabolism in the parietal lobe (Kotrla et al., 1995). Agitation was associated with atrophy of the frontal lobe, insula, amygdala, cingulate gyrus, and hippocampus (Trzepacz et al., 2013). The results of this study indicated that RT was not associated with the above neuropsychiatric symptoms. We will further explore the relationship between RT and other neuropsychiatric symptoms of AD, such as hallucination, illusion and delusion, in future work.

Finally, this study demonstrated that the ADL score in the abnormal AD-RT group was significantly increased, and there was a significant and negative correlation between the average RT of the right eye and the ADL score. The overall cognitive function, memory, attention, and executive function in the abnormal AD-RT group were severely damaged, which might result in significant impairment of ADL.

Despite the clinically interesting and potentially useful findings of this study, it has some limitations that should be noted. First, it was challenging to obtain the cerebrospinal fluid (CSF) from patients with AD who were elderly or who had either spinal deformities, bone hyperplasia, or other related conditions. The amyloid PET and/or fluorodeoxyglucose (FDG)-PET have not been covered by our medical insurance and are expensive for many families in China. Therefore, there was a lack of support for biomarkers (e.g., amyloid PET, CSF, and/or FDG-PET) in the criteria for the diagnosis of MCI due to AD or AD dementia adopted by this study. Second, in this study, RT assessed by OCT seemed not able to recognize patients with AD in almost 50% of the cases. In fact, more data on, for example, macular volume and vision are important regarding the visual recall and are pivotal for the results of cognitive tests and ADL. In the future, more data on the combined analyses of RT and GCCT need to be analyzed. Third, as half of our patients did not complete important MRI sequences, such as 3D-T1, diffusion tensor imaging, or functional MRI, the sample size was too limited to make robust conclusions from the neuroimaging assessments. Therefore, based on this study, we will undertake further research on the correlations between OCT values and neuroimaging results. Fourth, as a cross-sectional study, it is difficult to eliminate the effects of all the influencing factors. We are in the process of planning longitudinal studies that should be able to elucidate the progression of RT in patients with AD. Fifth, multiple comparisons may increase the probability of a type I error, especially in this study with a relatively small sample. However, the correction of multi-factor comparison may increase the probability of a type II error in this study. This is a preliminary exploration and large samples and the correction of multi-factor comparison are necessary in the future studies.

In conclusion, this study indicates that lower RT value may be correlated with cognitive impairment, and RT may serve as an indicator of cognitive decline in patients with AD. Further studies are required to confirm our findings.

## Data Availability Statement

The raw data supporting the conclusions of this article will be made available by the authors, without undue reservation.

## Ethics Statement

This study involving human participants was reviewed and approved by the ethics review board of Beijing Tiantan Hospital, Capital Medical University, Beijing. The patients/participants provided their written informed consents to participate in this study.

## Author Contributions

T-hL drafted the manuscript, carried out the analysis of data, accepted responsibility for the conduct of the research, final approval for the research, and performed the acquisition of data and the statistical analysis. ZJ drafted the manuscript, prepared the study design, carried out the analysis of data, accepted responsibility for the conduct of the research, provided final approval, and performed the acquisition of data and the statistical analysis. Y-zQ, W-jZ, and X-mW accepted responsibility for the conduct of the research and provided final approval. PG, D-yD, and D-nL carried out the acquisition of data, accepted responsibility for the conduct of the research, and provided final approval. H-yG drafted the manuscript, accepted responsibility for the conduct of the research, and provided final approval. L-xL carried out acquisition of data, accepted responsibility for the conduct of the research, and provided final approval. WZ prepared the study design, carried out the analysis of data, accepted responsibility for the conduct of the research, provided final approval, and performed the acquisition of data, the statistical analysis, and study supervision. All authors contributed to the article and approved the submitted version.

## Conflict of Interest

The authors declare that the research was conducted in the absence of any commercial or financial relationships that could be construed as a potential conflict of interest.
