# Supplementary material for: The Relationship Between Retinal Nerve Fiber Layer Thickness and Clinical Symptoms of Alzheimer's Disease
Source: Front Aging Neurosci. 2021 Jan 29;12:584244. doi: 10.3389/fnagi.2020.584244 (PMC7878673; doi:10.3389/fnagi.2020.584244)

Supplementary Material 1: OCT scans in normal subjects

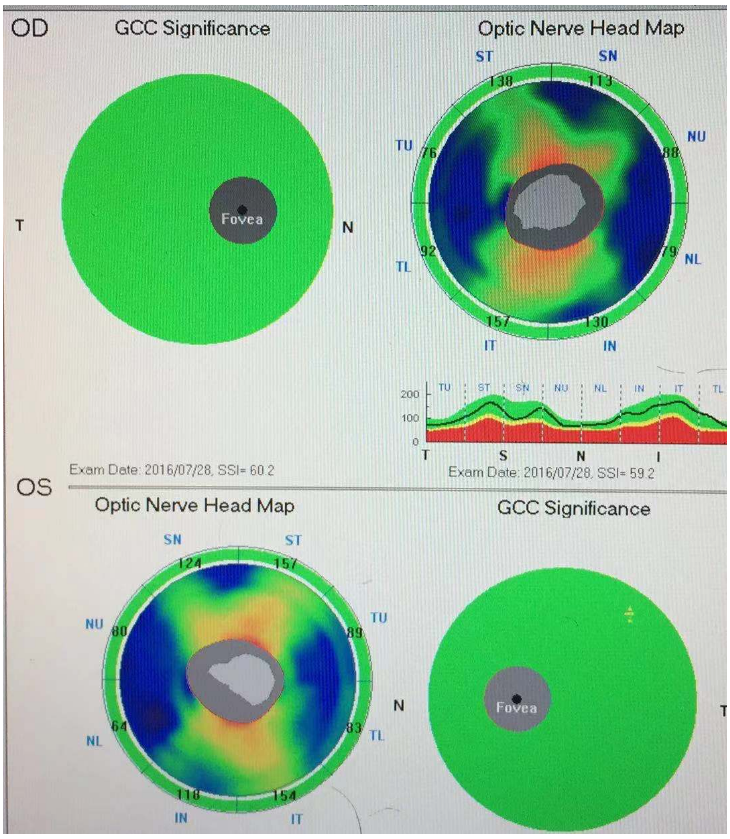

Supplementary Material 2: OCT scans in mild AD patients

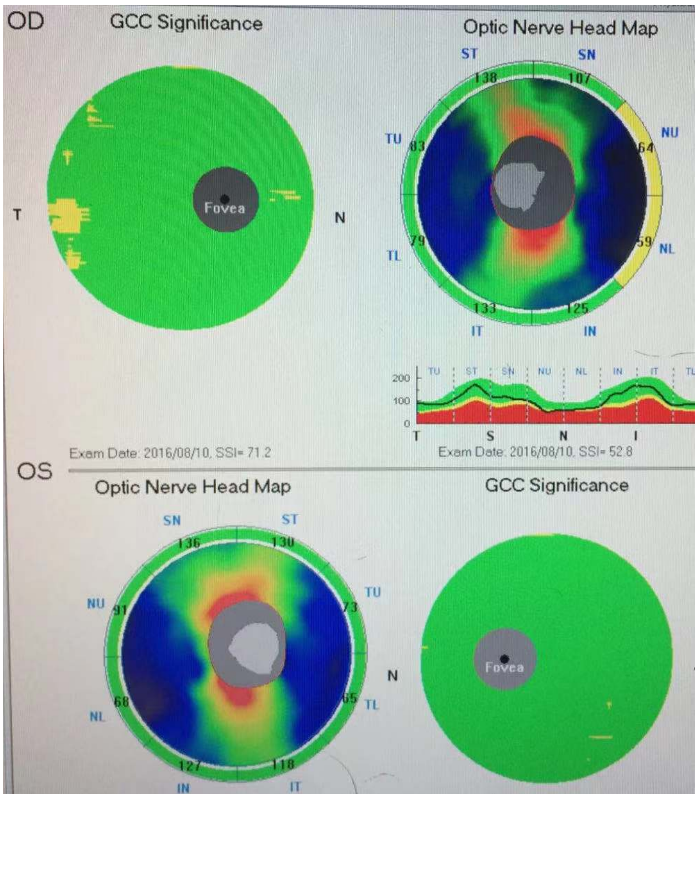

Supplementary Material 3: OCT scans in severe AD patients

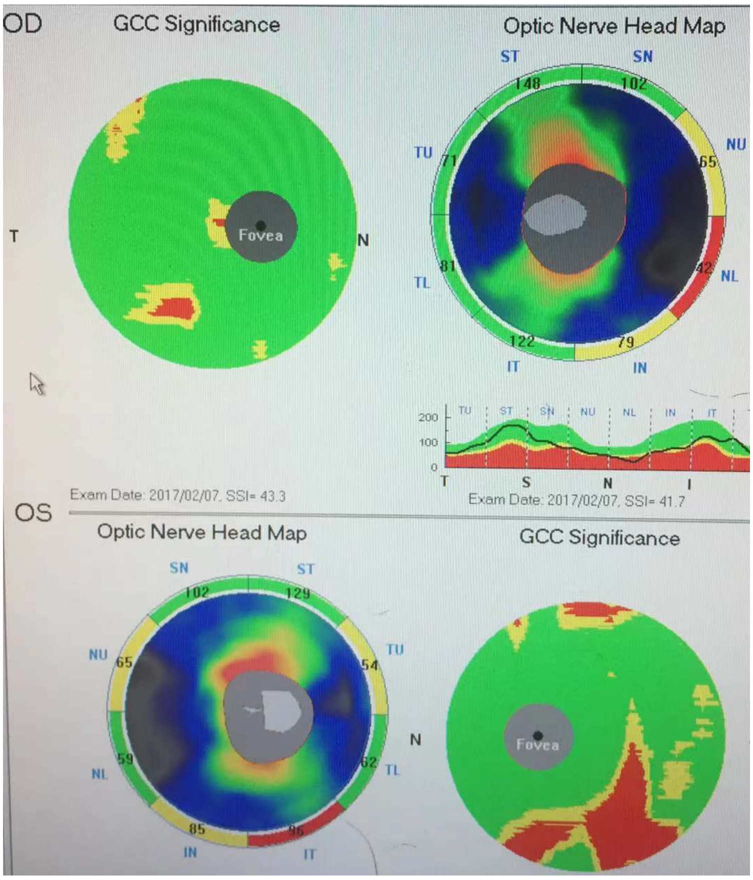

Supplement: Supplementary file 1 [file Image_1.PDF]
